# Context-Driven Ship, Vehicle, and Aircraft Detection in Colored Synthetic Aperture Radar (SAR) Images

**DOI:** 10.3390/s26175427

**Published:** 2026-08-27

**Authors:** Zhe Geng, Linyi Wu, Minjie Sun, Yu Zhang, Yuan Meng, Lujia Yao, Daiyin Zhu

**Affiliations:** College of Electronics and Information Engineering, Nanjing University of Aeronautics and Astronautics, Nanjing 211106, China; wly5010106@nuaa.edu.cn (L.W.); minjiesun@nuaa.edu.cn (M.S.); zy149480@nuaa.edu.cn (Y.Z.); mengyuan417@nuaa.edu.cn (Y.M.); 072220103@nuaa.edu.cn (L.Y.); zhudy@nuaa.edu.cn (D.Z.)

**Keywords:** synthetic aperture radar (SAR), deep learning, ship detection, vehicle detection, airplane detection

## Abstract

In slow-time colorized subaperture image (CSI), anisotropic targets that reflect strongly when viewed from specific angles appear in vivid colors, which makes them stand out against isotropic background that reflects energy uniformly across all angles. It leads to more accurate annotation labels for ships, vehicles, and airplanes in SAR images and better SAR automatic target detection (ATD) performance. Unfortunately, although many port-related CSI products collected by satellite-borne SAR systems are released for free public access and could be leveraged for ship detection research, those that could support vehicle and airplane detection are rare. To investigate performance improvement in deep learning-based SAR ATD that could be brought by colored SAR images, three novel SAR-ATD frameworks are proposed for ship, vehicle, and aircraft detection, respectively. (1) Context-guided ensemble learning (CGEL) is proposed for ship detection, where state-of-the-art high-resolution colorized spotlight SAR images are exploited to enhance the visual features of ships and reduce false alarms, while the potential ship berthing/docking areas are delimited with adaptive intensity shading (AIS). (2) Context-driven SAR image recoloring and enhancement mechanism (CD-SAR-REM) is proposed to generate a context-driven color-enhanced version of the original SAR image based on AIS so that potential parking regions are highlighted. (3) Color feature-aided aircraft detection. In case that CSI products are unavailable, pseudo-color SAR images are generated based on phase congruency and the contextual information extracted by the segmentation module is used to refine the initial predictions generated by the core detection network. Experimental results show that the performance of the proposed context-driven ship, vehicle, and aircraft detection methods based on colored SAR images are superior to many state-of-the-art SAR ATD models.

## 1. Introduction

Ship, vehicle and airplane detection are typical targets of interest for defense, intelligence, multi domain awareness, and disaster response. Although the images acquired by electro-optical (EO) and infrared (IR) sensors are more comprehensible to human operators, the former is only available when the light condition is favorable, while the latter suffers from noticeable performance degradation in the presence of thick water fog made of large droplets. Specifically, short-wave infrared (SWIR) has a wavelength of 1.4–3 μm and behaves similarly to visible light. Since SWIR wavelengths are smaller than most fog droplets, it generally fails in dense fog. Mid-wave infrared (MWIR) works with a wavelength of 3–5 μm and captures both reflected sunlight and thermal emission. Long-wave infrared (LWIR) works at a wavelength of 8–14 μm and can see in total darkness by measuring emissivity. Since the LWIR wavelengths are greater than most fog droplets, it is less affected by dense fog than SWIR and MWIR. However, LWIR fares differently in pollution fog made of PM2.5 and PM10 and normal water fog. In the former case, LWIR can maneuver through the tiny solid particles with ease and obtain perfect IR images since no absorption occurs. In the latter case, large amount of infrared energy could be absorbed by the atmospheric moisture if the target of interest is hundreds of meters away. Thermal washout can also happen if the fog is very thick, which leads to a gray blurred image with no temperature difference. In a typical foggy cold winter night, which is the worst possible case, the contrast of infrared image is expected to degrade severely due to isothermal equilibrium, low radiative energy, and heavy absorption. In this case, synthetic aperture radar (SAR) system, which can penetrate fog/smoke/clouds easily, becomes the last resort.

Although SAR automatic target detection (ATD) is essential for all-day all-weather ground surveillance, it involves more technical complexities than object detection in aerial images. First, ship, vehicle, and plane detection in SAR images share some common challenges, which originate from the special imaging mechanism of SAR system which defies the Gestalt rule. To begin, the movement of the platform is used to mimic a large antenna with super-resolution. Yet, the movement can never be perfect, not to mention the approximations made in the imaging forming process. Moreover, each component of the target has a fluctuating radar cross section (RCS), which results in a SAR signature with fluid nature. As a result, SAR images corresponding to the same object could exhibit completely different appearances depending on the circumstances. That said, ship, vehicle, and plane in SAR image exhibit distinctive characteristics, hence demanding different strategies.

Due to diversity of imaging distance, orientation and ship tonnage, ship targets have huge intra-class size variations, ranging from dozens of pixels to hundreds of pixels. According to a review paper [1], by 2022 the mAP@50 had increased to 97.8% [2] on the SAR Ship Detection Dataset (SSDD) [3], the first large-scale SAR ship detection dataset free for public access. Meanwhile, the AP reached 94.46% [4] on SSDD+, the first dataset for SAR ship detection with an oriented bounding box. The other widely used benchmark SAR ship detection datasets until 2022 include SAR-Ship-Dataset [5], AIR-SARShip1.0/AIR-SARShip2.0 [6], HRSID [7], LS-SSDD-v1.0 [8], SRSDD-v1.0 [9]. In 2024–2025, SARDet100K [10], which contains more than 70,000 SAR images of ships sourced from the ship detection datasets mentioned above, and the matching rotated object detection dataset RSAR are released by researchers from Nankai University [11]. In 2025, a fine-grained SAR dataset covering 7 subcategories of ship targets, which include bulk carriers, warships, etc., is released by Wu et al. from the Chinese Academy of Sciences [12]. In 2016, Sun et al. from Nankai University constructed a new multiclass fine-grained SARship dataset instance-level SARship classification (ISSC), based on which CNN-based architectures (e.g., FCOS [13], FoveaBox [14]), Vision Transformer (ViT) [15], Swin Transformer (Swin-T) [16], and pyramid vision transformer (PVT) [17]-based backbones integrated into the RetinaNet framework (i.e., RetinaNet_ViT, RetinaNet_Swin-T and RetinaNet_PVT), as well as recent state-of-the-art SAR-specialized detectors (LiteSAR-Net [18]) are tested. Experimental results show that the overall detection accuracy on the ISSC dataset ranging from 23.8% to 40.9% in mAP. Despite the significant progress made in the field of SAR ship detection in recent years [19], research in context-guided ship detection with high resolution CSI products is rare.

Vehicles are relatively small compared to background clutters, and the intra-aspect difference of the same target could be greater than intra-class difference due to anisotropic scattering. In [20], Zou et al. considered the problem of vehicle detection based on semantic-context enhancement (SCE), and proposed SCEDet, which consists of a SCE module to preserve the target details and the global context information, a multiscale semantic feature (MSFF) module to balance the high-level semantic features and target details, and a global context enhancement (GCE) block to aggregate the context information from different scales. In [21], a cross-sensor SAR target detection method based on semantic scattering graph structure alignment (SSGSA) is proposed, where sampled semantic scattering points are transformed into graph structures, enhanced with Graph Convolutional Network (GCN), and aligned at levels of node feature and local feature in cross-sensor tasks. Experiments based on FARAD-Ka (FK), FARAD-X (FX) and Ku-band MiniSAR (M) vehicle detection dataset show that the proposed method achieves an mAP of 73.3% in FK→M task, which is 30–40% higher than the classic deep learning algorithms, and an mAP of 75.4% in FX→M task, which is 14% higher than the algorithm proposed by Hsu et al. in [22].

Aircraft often manifest as highly aspect/pose-sensitive, disjointed blobs made of pixels with fluctuating levels of brightness, which thwarts the development of deep learning-based SAR aircraft target detection and recognition algorithms. By far, most studies in this field are based on SAR images collected by Gaofen-3, HiSea-1, or TerraSAR-X, such as MSAR dataset [23], SAR aircraft detection dataset (SADD) [24], and SAR-AIRcraft-1.0 [10,25]. It is shown in [21] that, although the SSGSA method exhibits excellent performance on vehicle detection tasks, when the algorithm is applied to the Gaofen-3 (GF) and HiSea-1 (HS) SAR airplane detection dataset, mAP in the GF-HS task is only 48.4%. It indicates that the performance of SAR plane detection is currently limited by the quality of images collected more than 10 years ago by legacy SAR satellites. In [26], Zhang et al. introduced a novel scattering feature relation enhancement network (SFRE-Net), which consists of a cascade transformer block (TRsB) structure, a feature-adaptive fusion pyramid structure (FAFP), and a context attention enhancement module (CAEM). Experiments based on the Gaofen-3 dataset show that SFRE-Net exhibits better performance than classic CNN architectures such as CenterNet [27] and YOLOX [28]. In [29], Huang, et al. proposed the physics-guided detector (PGD) learning paradigm for SAR aircraft detection, which consists of three key modules: physics-guided self-supervised learning (PGSSL) that is suitable for both CNN and transformer architectures, physics-guided feature enhancement (PGFE) that is compatible with arbitrary feature pyramid network (FPN)-style neck, and physics-guided instance perception (PGIP) to separate dominant scattering points from the secondary ones for fine representation learning. The PGD network achieved 90.7% mAP on the benchmark dataset SAR-AIRcraft-1.0 covering 7 aircraft categories. Considering that SAR aircraft targets lack consecutive contours, Dou et al. [30] proposed to incorporate shape prior knowledge for SAR aircraft reconstruction. Similarly, Geng et al. proposed to estimate the dimension/shape/contour of the aircraft based on the information extracted from infrared images [31]. Sun et al. proposed a SAR-to-optical translation network as a pre-processing procedure to facilitate both automatic and manual aircraft recognition within the framework of conditional generative adversarial network (cGAN) [32]. In [33], pseudocolor SAR image is generated through speckle suppression and channel combination. Experiment results based on a dataset containing GaoFen-3 SAR satellite images featuring 12 categories of aircraft show that the channel combination method leads to an increase of 4.5% in mean average precision (mAP), while the despeckle algorithm improves the mAP by 2.7%.

The launch of Colorized SAR Image (CSI) products by commercial SAR companies-including ICEYE, Capella Space and Umbra-since 2024, is a big game changer for SAR image interpretation. CSI could be classified as fast-time CSI and slow-time CSI. Fast-time CSI highlights the frequency dependence of the target of interest. The easiest way to create fast-time CSI product is to divide the bandwidth into low-frequency, mid-frequency, and high-frequency sub-bands and designate the data to red, green, and blue channels, respectively. In this case, if an object appears red, it indicates the object has a higher RCS at lower frequencies. Slow-time CSI reflects the aspect sensitivity of the target of interest. A straightforward slow-time processing method is to assign images generated from early, center and late observation angles to red, green, and blue channels. As a result, anisotropic targets that reflect strongly when viewed from specific angles appear in vivid colors. For example, if certain component of the target reflects strongly only when the radar is at the beginning of its path, that part will appear bright red. Although the baseline CSI processing methods mentioned above could colorize SAR images, the resulting resolution could be three times coarser than the original and lead to “rainbow noise” effect since the speckle in these channels are uncorrelated. Therefore, modern CSI systems often employ multi-tap sliding window and multi-chromatic analysis (MCA). For fast-time CSI, “hyper-spectral” radar cube is created by sliding the window across the full spectrum in small steps, which is then collapsed into RGB with the leading 1/3, the middle 1/3, and the lagging 1/3 contributing the red, green, and blue, respectively. For slow-time CSI, a stack of sub-aperture images are created by sliding the window across the observation angles. The hue, saturation, and value of each pixel is determined by the centroid, variance, and total energy reflected in slow-time “energy vs. angle” curve, respectively. By employing an overlap ratio of 25–50%, the color info is preserved while the resolution loss is limited.

To investigate performance improvement in deep learning-based SAR ATD that could be brought by colored SAR images, context-guided ensemble learning (CGEL) is proposed for ship detection. A SAR ship detection dataset is constructed based on the state-of-the-art high-resolution colorized spotlight SAR images released by commercial SAR companies [34,35,36], which provides both horizontal bounding boxes (HBB) and orientated bounding box (OBB) ground-truth annotations. To separate the potential ship berthing/docking area from the irrelevant background regions while preserving the contextual information, land–water masking is carried out with adaptive intensity shading (AIS). Considering that each model has its unique inherent bias that brings favorable advantage over certain types of image samples and disastrous mistakes against other types of data, a multi-model ensemble learning strategy based on oriented weighted box fusion (OWBF) is proposed.

Although many port-related CSI products from SAR satellites could be used for ship detection, those could support vehicle and airplane detection are rare. Therefore, a context-driven SAR image recoloring and enhancement mechanism (CD-SAR-REM) is employed for color coding and vehicle detection. To fully exploit the prior probability of the vehicle distribution pattern contained in the scene context, the potential parking regions are identified by exploiting the cross-modality features of aligned SAR optical images based on AIS, while outputs generated by different types of SAR speckle filters are plotted with different colormaps and combined linearly to form RGB images for edge and texture feature extraction.

Since aircraft often manifest as highly aspect/pose-sensitive, disjointed blobs made of pixels with fluctuating levels of brightness, it is necessary to distinguish fragments of SAR signatures of aircraft from strong discrete clutters densely distributed in a typical airport. In case that CSI products are available, context-guided plane detection is carried out according to the global–local–target (GLT) workflow, which involves SAR optical image registration, semantic segmentation of runway and apron based on AIS, and aircraft detection with information-sharing between OPTimal and suboptimal image slices (OPTshare). In case that CSI products are unavailable, pseudo-color SAR images are generated based on phase congruency (PHC) by extracting the local spatial phase and structure orientation of the image, while the contextual information extracted by the post-processing multi-space clustering constraint (MSCC) module is used to refine the initial predictions generated by the core detection network based on the image segmentation result in $L^{*}a^{*}b^{*}$ color space.

This work extends our previous works on context-guided SAR ship [19], vehicle [31,37], and aircraft detection [38] to the domain of color-coded SAR images. The major contributions of this work are summarized as follows.

(1) To deal with ship size diversity and inshore complex background, CGEL strategy is proposed for ship detection, where AIS-based land–water masking is carried out and multi-model ensemble learning strategy is employed.
(2) CD-SAR-REM is proposed to generate context-driven color-enhanced SAR images by combining the color-coded parking-highlighted images generated by diverse speckle filters with the original SAR image based on AIS.
(3) CSI-based aircraft detection based on the GLT workflow is proposed. In case that CSI products are unavailable, pseudo-color SAR images are generated based on PHC and the contextual information extracted by the segmentation module is used to refine the initial predictions generated by the core detection network.

The rest of this paper is organized as follows. Section 2 details the CGEL strategy proposed for ship detection. Section 3 presents CD-SAR-REM for SAR image color coding and feature enhancement. Section 4 describes the proposed GLT work flow as well as the PHC-based pseudo-color SAR image generation method for color feature-aided aircraft detection in case that CSI products are unavailable. Section 5 presents the experimental settings and the experimental results. Section 6 concludes the full paper.

## 2. CGEL for Ship Detection

**Figure 1:**
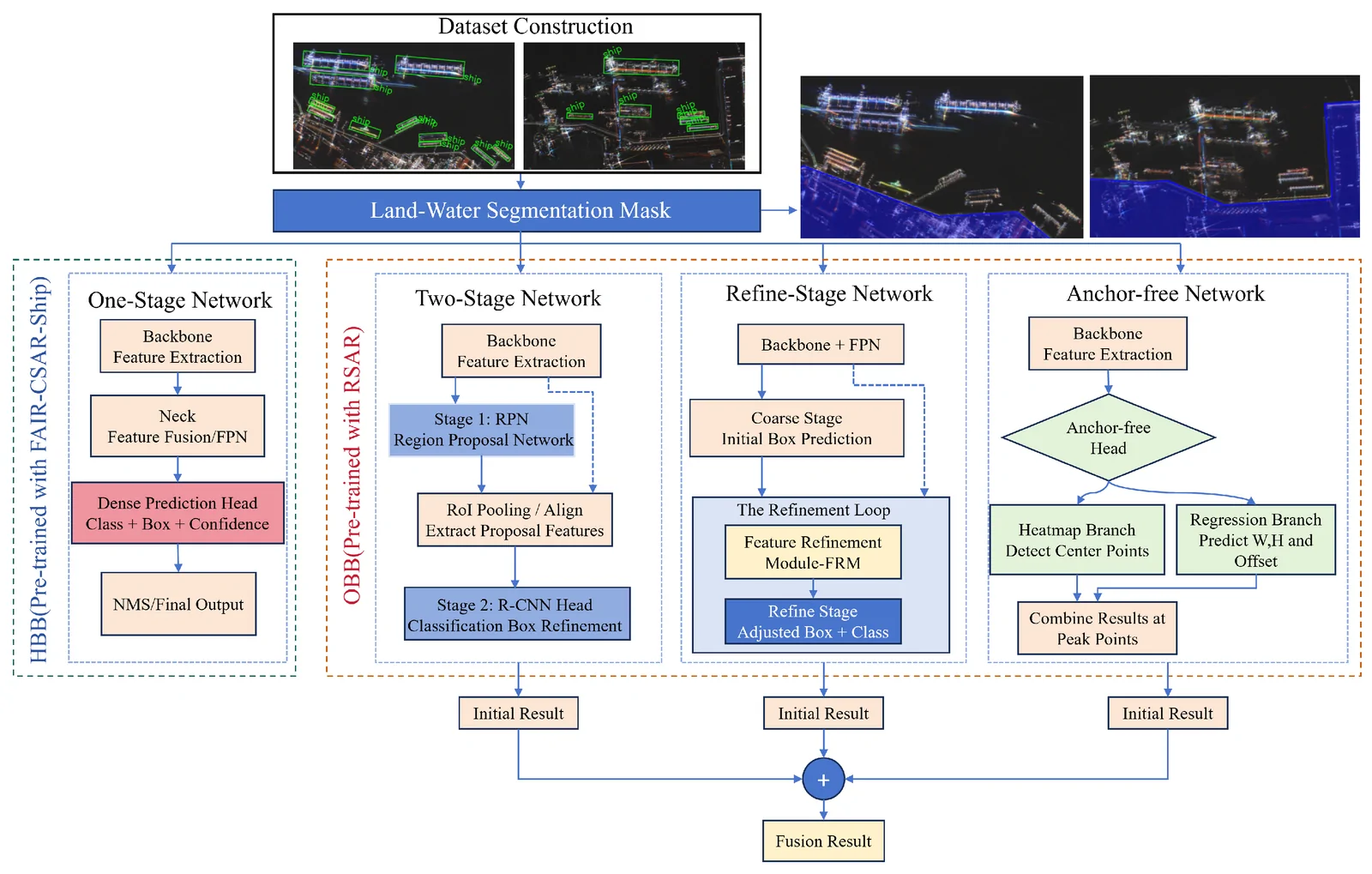
CGEL for SAR ship detection, which incorporates CSI-based ship annotation, AIS-based land–water segmentation, as well as ensemble learning based on two-stage, refine-stage and anchor-free OBB models.

To enhance the visual features of ships and reduce false alarms, the potential ship berthing/docking areas are delimited by utilizing the stable context information contained in the optical image acquired in favorable light conditions. To pinpoint the potential ship berthing/docking area while preserving the contextual information, such as land and quayside resources that could provide hints on the handling capacity of the port terminal, land–water masking is carried out with AIS based on clustering-based spatial topological constraint rather than global thresholding, which could black out all the land regions. Figure 1 illustrates the CGEL flowchart, which is motivated by the fact that multi-model ensemble learning has been known to be effective in overcoming inherent biases of individual models employing different design principles (e.g., one-stage, two-stage, anchor-free and refine-stage models). Considering that most of the existing SAR ship detection datasets contain grayscale SAR images (e.g., FAIR-CSAR-Ship dataset for HBB and RSAR dataset for OBB), the CSI products are used in color to facilitate image labeling but are converted to grayscale images for network training and test so that pretrained models can be fully exploited. As mentioned before, modern CSI employed by commercial satellite SAR companies like Capella Space and ICEYE is based on ultra-spotlight mode with extra-long dwelling time, multi-tap sliding window, and MCA. It is not only a process that could bring color to SAR image but is also effective in speckle noise elimination. Moreover, for two or three ships docked side-by-side at the port terminal, the edges appear to be clearer due to obvious color change so that they would not be mistaken as one ship. Finally, following the logic of “human-in-loop” (human vision deals with multichannel images better as color representations), the prediction results are synchronized to the color SAR image for human operators to inspect and analyze.

The oriented weighted box fusion (OWBF) method is introduced to fuse the initial predictions made by two-stage, refine-stage and anchor-free OBB models. Consider OBB with five parameters (x,y,w,h,θ), with (x,y) representing center coordinates, and w,h,θ representing width, height, and rotation angle, respectively. First, sort the oriented bounding boxes based on the confidence scores from high to low and discard those at the bottom. After that, spatial clustering is implemented based on the polygon IoU based on a predetermined threshold. Once the clustering process is completed, spatial reconstruction and smoothing are carried out for (x,y,w,h,θ) of the prediction bounding boxes belonging to the same cluster by jointly considering the historical performance and the current prediction score. The reconstructed bounding box $B^{*}$ is given by

(1) $$\begin{array}{c} B^{*}=\frac{\sum_{k=1}^{K}S_{k}\cdot W_{k}\cdot B_{k}}{\sum_{k=1}^{K}S_{k}\cdot W_{k}} \end{array}$$

with a confidence score of

(2) $$\begin{array}{c} S^{*}=\frac{1}{K}\sum_{k=1}^{K}S_{k} \end{array}$$

where *K* is the total number of orientated prediction bounding boxes in the existing cluster, $B_{k}=\left(x_{k},y_{k},w_{k},h_{k},\theta_{k}\right)$ is the parameters of the *k*-th prediction bounding box, $S_{k}$ is the initial confidence score of the *k*-th prediction bounding box, and $W_{k}$ is the weight for the corresponding baseline model.

## 3. CD-SAR-REM for Vehicle Detection

Unlike close-up images taken by cameras, remote sensing images are often available in Geotiff format, which provides accurate Longitude/Latitude information, based on which SAR optical image registration could be carried out based on images acquired from open platforms such OpenStreetMap and GoogleEarth. In our previous work [39], it has been demonstrated that segmentation results generated based on aligned SAR optical images are close to ground truth than those generated based on single-modality images, especially in the presence of moderate–heavy fog. In our previous works, the semantic masks corresponding to roads and parking lots were applied directly to the SAR images to suppress false alarms in SAR vehicle detection [40]. Although some promising preliminary results have been obtained, they did not account for the mismatch between SAR and optical images due to continual changing of the landscape, i.e., dynamic context. First, the development of modular integrated construction (MIC), which limits on-site activity to the final assembly and installation phases that could be finished in hours, makes it possible for the landscape to change within hours. In some extreme cases, such as military conflicts, earthquake, flood, etc., the landscapes that are used as context could change in a blink. In a typical earthquake, the buildings become unstable features while the ground track field remains the relatively stable feature. In contrast, the ground track field is the first to disappear in the typical scenario of flash flood. The same rule applies to military conflicts, during which the change of role among the land features becomes unpredictable.

To cope with dynamic context, CD-SAR-REM is proposed in this work, which aims to generate a knowledge-aided color-enhanced version of the original SAR image by first constructing a knowledge-aided masking image, $I_{mask}$, and then combine it with the original SAR image, $I_{SAR}$ based on AIS. $I_{mask}$ is created by first removing non-parking regions based on the semantic mask and then combining the complementary features extracted by different speckle filters. Finally, the context-driven color-enhanced images used for vehicle detection, $I_{context}$, are obtained by combining $I_{SAR}$ and $I_{mask}$ linearly as $I_{context}\left(\alpha ,\beta \right)=\alpha I_{mask}+\beta I_{SAR}$, where α and β are weighting factors that could be adjusted. Four representative speckle filters are considered: IDAN filter [41], refined Lee filter [42], improved Lee Sigma filter [43] and boxcar (mean) filter [42].

The IDAN filter [41] forms adaptive neighborhood by testing the pixel one by one and aggregating the pixels if they belong to the same statistical population as the seed pixel. The Refined Lee filter [42] belongs to the family of minimum mean squared error (MMSE) filter, and it implements piecewise filtering based on the variance of intensity, $\sigma^{2}$. In case that $\sigma^{2}$ is less than a predetermined threshold $\sigma_{tr}^{2}$, the region is smoothed and output is the local mean. Otherwise, it is presumed to be edge area, in which case overlapping windows are applied to suppress the speckle noise while preserving the edge feature. The improved Lee Sigma filter [43] is a modified version of the conventional Lee sigma filter, and its core intention is to preserve highly reflective point targets. Specifically, the center pixel in a sliding window is filtered based on the average of pixels within the two-sigma range as the conventional Lee sigma filter does; however, if the number of pixels that have a value greater than the 98th percentile meets a user-defined threshold, all of the pixels are preserved. The boxcar (mean) filter [42] assigns to each pixel the average of the neighbouring pixels, which makes it a good fit for homogeneous areas but could lead to blurred details.

**Figure 2:**
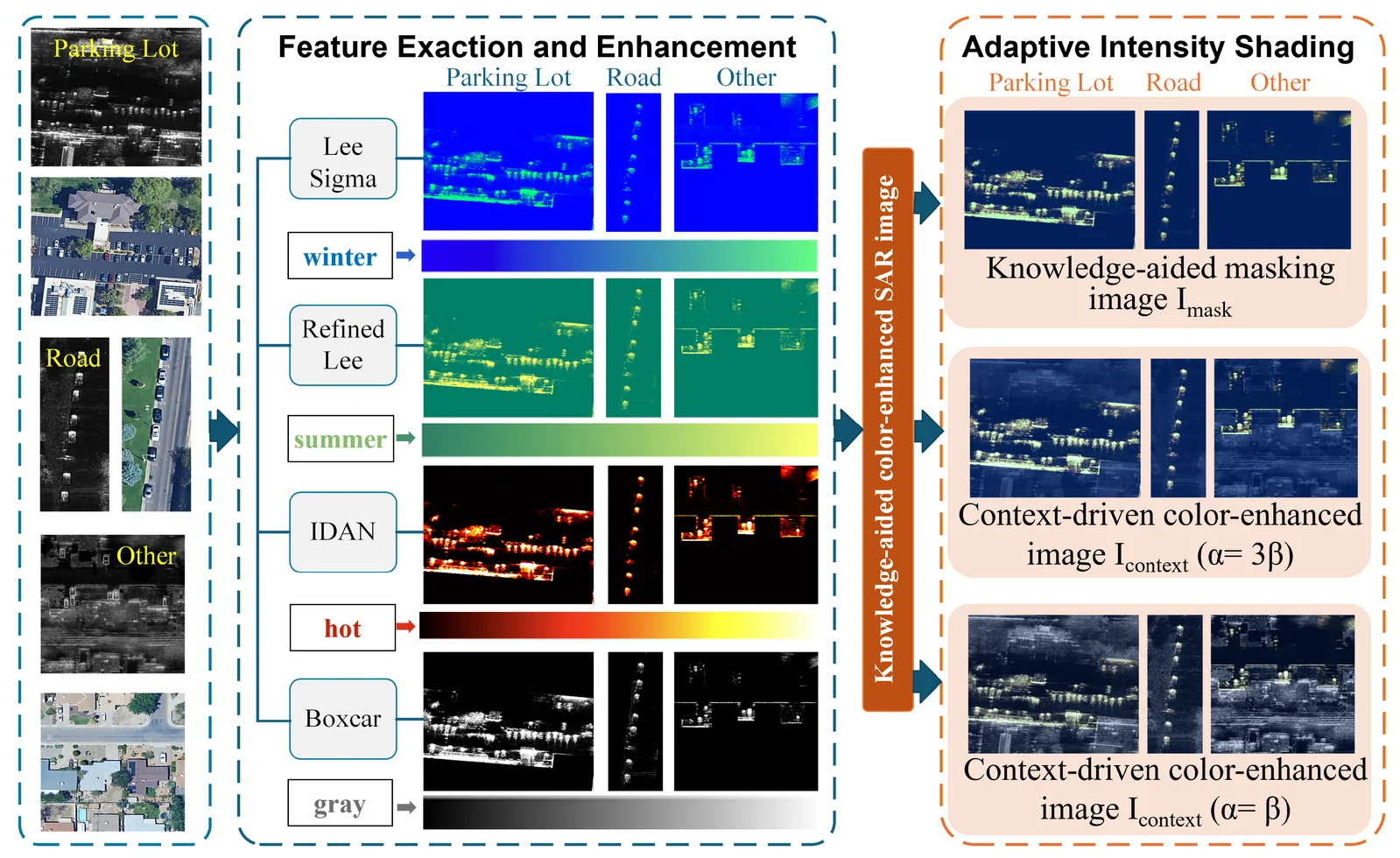
CD-SAR-REM to generate a knowledge-aided color-enhanced version of the original SAR image based on AIS so that potential parking regions are highlighted.

Figure 2 illustrates the SAR image enhancement workflow with three representative aligned SAR optical images pairs corresponding to parking lot, road and sparse parking (other). The knowledge-aided masking images, $I_{mask}$, are obtained by first removing non-parking regions based on the semantic mask and then combining the outputs of four types of filters that are color-coded with different colormaps so that the complementary features are jointly exploited. The context-driven color-enhanced images $I_{context}$ shown on the right side of Figure 2 are obtained by combining $I_{SAR}$ and $I_{mask}$ linearly by setting α=3β and α=β, respectively.

## 4. Color Feature-Aided Aircraft Detection

### 4.1. CSI-Aided Aircraft Detection Based on GLT Workflow

**Figure 3:**
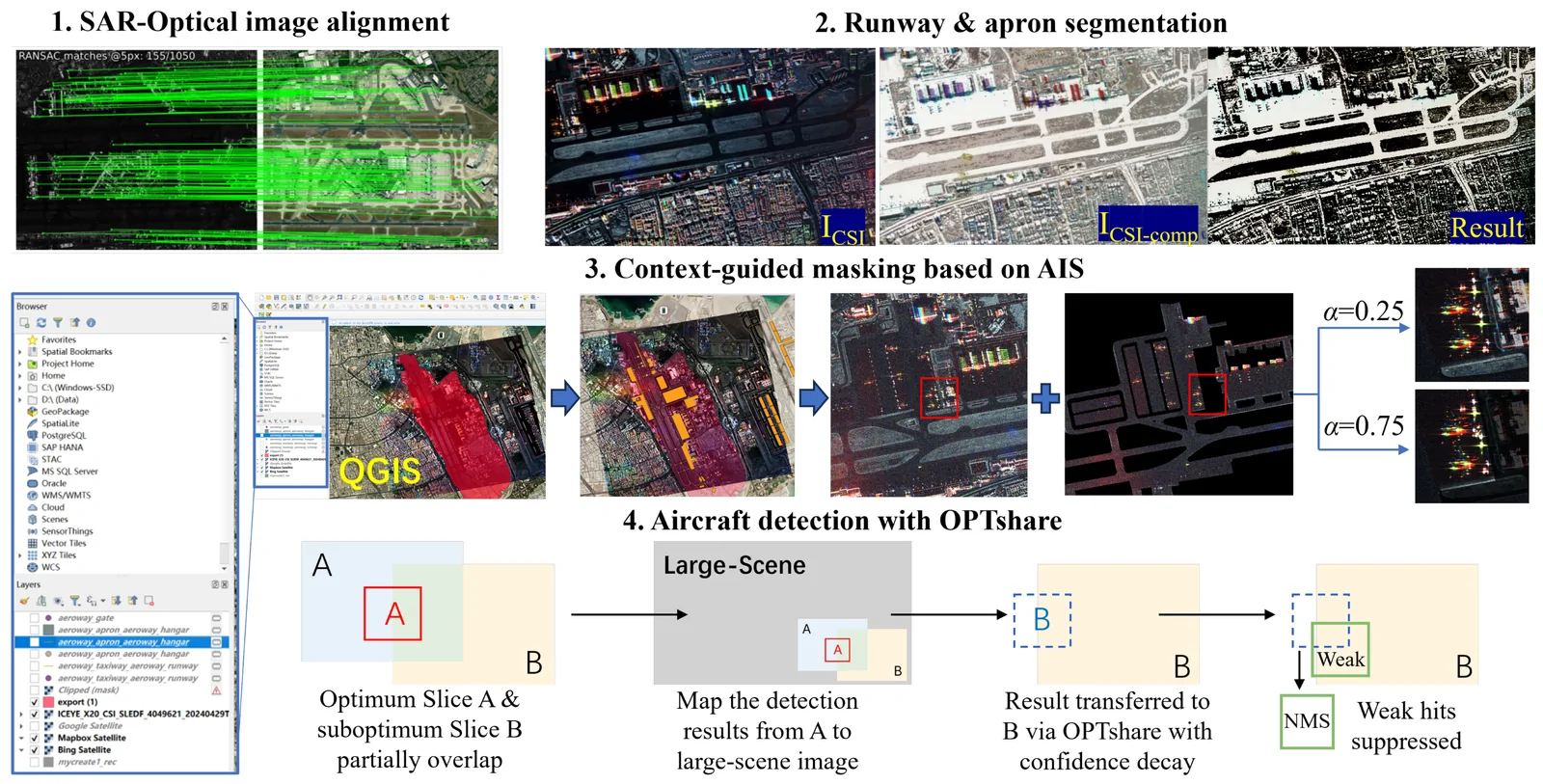
Aircraft detection in large-scene SAR images with GLT, which includes SAR optical image registration (Step 1), semantic segmentation of runway and apron (Step 2), context-guided masking based on AIS (Step 3, QGIS 3.38.2 was used), and aircraft detection based on OPTshare (Step 4).

Since aircraft is much larger than vehicles in size, SAR aircraft detection based on satellite SAR images is technically feasible. When CSI product is available, the GLT workflow illustrated in Figure 3 could be followed. First, SAR optical image alignment could be conducted with deep learning solutions such as MapGlue [44]. After that, leveraging the fact that aircraft are usually parked on runways or taxiways made of smooth asphalt or concrete, which appear pure black in CSI products, K-means clustering in the $L^{*}a^{*}b^{*}$ color space could be performed, based on which segmentation masks are generated. The ICEYE CSI product corresponding to Hamad International Airport in Qatar ($I_{CSI}$) is used as an illustrating example, with $I_{CSI-comp}$ formed by taking the complementary colors. The images below $I_{CSI}$ and $I_{CSI-comp}$ are the results generated by Matlab^®^ 2025b Color Thresholder toolbox.Following image segmentation, context-guided masking based on AIS is implemented so that false alarms induced by discrete clutters in regions that are unlikely for aircraft to be parked are suppressed while the contextual information is preserved. Two examples are provided in Figure 3 for α=0.25 and α=0.75, where 0≤α≤1 is the weighting factor applied to the mask and α=1 corresponds to keep only the known aprons and runways while removing all other regions. Finally, aircraft detection is conducted based on OPTshare. Suppose Slice A and B are the optimum and the suboptimum slices, respectively, and are partially overlapped. If the overlap ratio is greater than a predetermined threshold, $Tr_{overlap}$ The detection results from A are mapped to large-scene images and then transferred to Slice B with a confidence decay of factor. The “ghost detections” or weak hits from the neighboring suboptimal slices (OPTshare) are then suppressed with non-maximum suppression (NMS).

### 4.2. Context-Guided Aircraft Detection with Colored SAR Images Based on PHC

**Figure 4:**
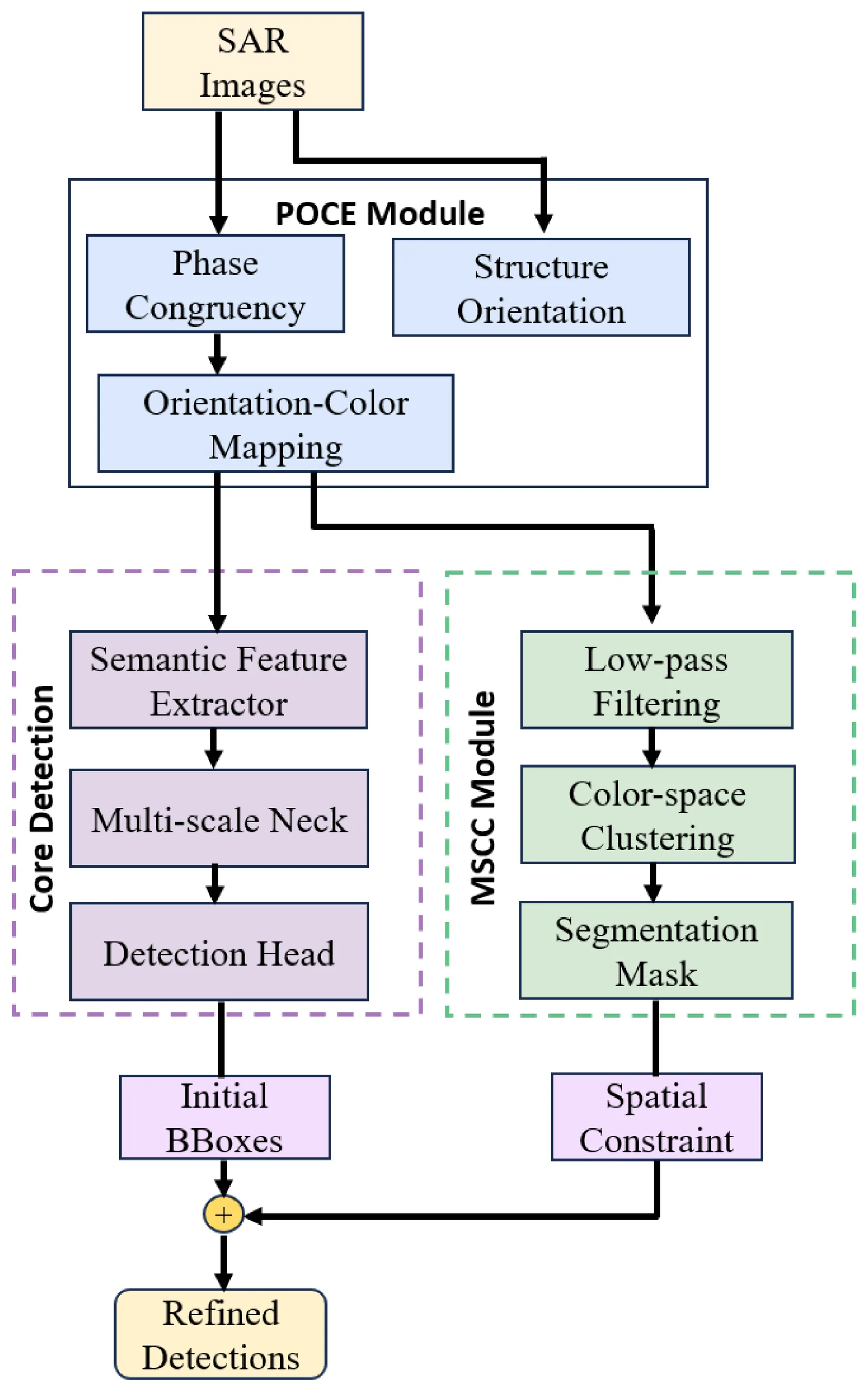
Pseudo-color SAR images creation based on PHC [45].

Figure 4, which is a modified version of the flowchart of PACE-Det [45], illustrates the process of pseudo-color SAR image based on PHC, which consists of three key modules: the phase-orientation color encoder (POCE) module to generate pseudo-colored SAR images based on PHC and structure orientation, the core detection network to generate initial predictions, and the MSCC post-processing module to refine the initial predictions based on the image segmentation result in $L^{*}a^{*}b^{*}$ color space.

#### 4.2.1. POCE for Pseudo-Color SAR Image Creation

First, Log-Gabor filter bank is used to decompose the SAR image into amplitude and phase components, based on which structural intensity could be calculated. After that, inverse Fourier transform is applied to obtain even-symmetric component $I_{scale,ori}^{even}$ and odd-symmetric component $I_{scale,ori}^{odd}$ so that the local amplitude is defined as

(3) $$\begin{array}{c} A_{scale,ori}\left(x,y\right)=\sqrt{I_{scale,ori}^{even}{\left(x,y\right)}^{2}+I_{scale,ori}^{odd}{\left(x,y\right)}^{2}} \end{array}$$

The PHC map can be calculated as

(4) $$\begin{array}{c} \mathrm{PHC}\left(x,y\right)=\frac{\max(E\left(x,y\right)-N_{T},0)}{\sum_{ori}\sum_{scale}A_{scale,ori}\left(x,y\right)+\epsilon } \end{array}$$

where $N_{T}$ is the adaptive noise threshold, E(x,y) is the summation of even-symmetric and odd-symmetric components across all scales and all orientations, and ϵ is a small constant to avoid division-by-zero errors. The orientation θ is obtained by applying an isotropic Gaussian smoothing kernel $G_{\sigma }\left(x,y\right)$ to the original gradient matrix for image $I_{SAR}$, in which process a robust structure tensor J is obtained as

(5) $$\begin{array}{c} J=G_{\sigma }⊛\left[\begin{array}{cc} I_{x}^{2} & I_{x}I_{y}\\ I_{x}I_{y} & I_{y}^{2} \end{array}\right]=\left[\begin{array}{cc} J_{xx} & J_{xy}\\ J_{xy} & J_{yy} \end{array}\right], \end{array}$$

where ⊛ denotes convolution, $I_{x}$ and $I_{y}$ are partial derivatives of $I_{SAR}$. The structure orientation (STO) feature is obtained as

(6) $$\begin{array}{cc} F_{STO}\left(x,y\right) & =\frac{\theta (x,y)\;\mathrm{mod}\;\pi }{\pi } \end{array}$$

(7) $$\begin{array}{cc} \theta (x,y) & =\frac{1}{2}\arctan\left(\frac{2J_{xy}}{J_{xx}-J_{yy}}\right) \end{array}$$

For R, G, B channels centered at predefined phase angles $\mu_{R}$, $\mu_{G}$ and $\mu_{B}$, the algorithm first calculates the angular distance between the local $F_{STO}\left(x,y\right)$ and the channel centers and then generates weight matrices accordingly.

#### 4.2.2. Core Detection Network

The loss function for the core detection network is defined as

(8) $$\begin{array}{c} L=L_{d}+\lambda_{PA}L_{PA} \end{array}$$

where $L_{d}$ represents the standard object detection loss made of two parts: classification loss and bounding box regression loss. $L_{PA}$ is the phase-aware alignment loss defined as the mean squared error (MSE) between the low-level PHC map PHC and the deep feature map $R(r_{i})$, which is given by

(9) $$\begin{array}{c} L_{PA}=\frac{1}{Q}\sum_{i}{∥R(r_{i})-\mathrm{PHC}∥}_{2}^{2} \end{array}$$

where *Q* is the number of elements in the feature map. $\lambda_{PA}$ in (8) is a weighting factor to be optimized based on the mission at hand and the backbone architecture. Without loss of generality, $\lambda_{PA}=1$ could be employed.

#### 4.2.3. MSCC for Prediction Refinement Based on Contextual Information

MSCC utilizes K-means clustering to segment aprons and runways, so that the false alarms induced by discrete clutters in unlikely aircraft parking regions are suppressed. Define *N* as the total number of image pixels, and $v_{i}\in R^{3}$ as the feature vector of the *i*-th pixel in the $L^{*}a^{*}b^{*}$ color space. The clustering algorithm aims to partition the feature space into *K* disjoint clusters $P=\{P_{1},P_{2},...,P_{K}\}$. The optimization objective function is given by

(10) $$\begin{array}{c} \arg\min_{P}\sum_{k=1}^{K}\sum_{v_{i}\in P_{k}}{∥v_{i}-\gamma_{k}∥}_{2}^{2} \end{array}$$

where $\gamma_{k}$ is the centroid vector of the *k*-th cluster [45].

## 5. Experimental Results

### 5.1. Ship Detection Experiment

**Figure 5:**
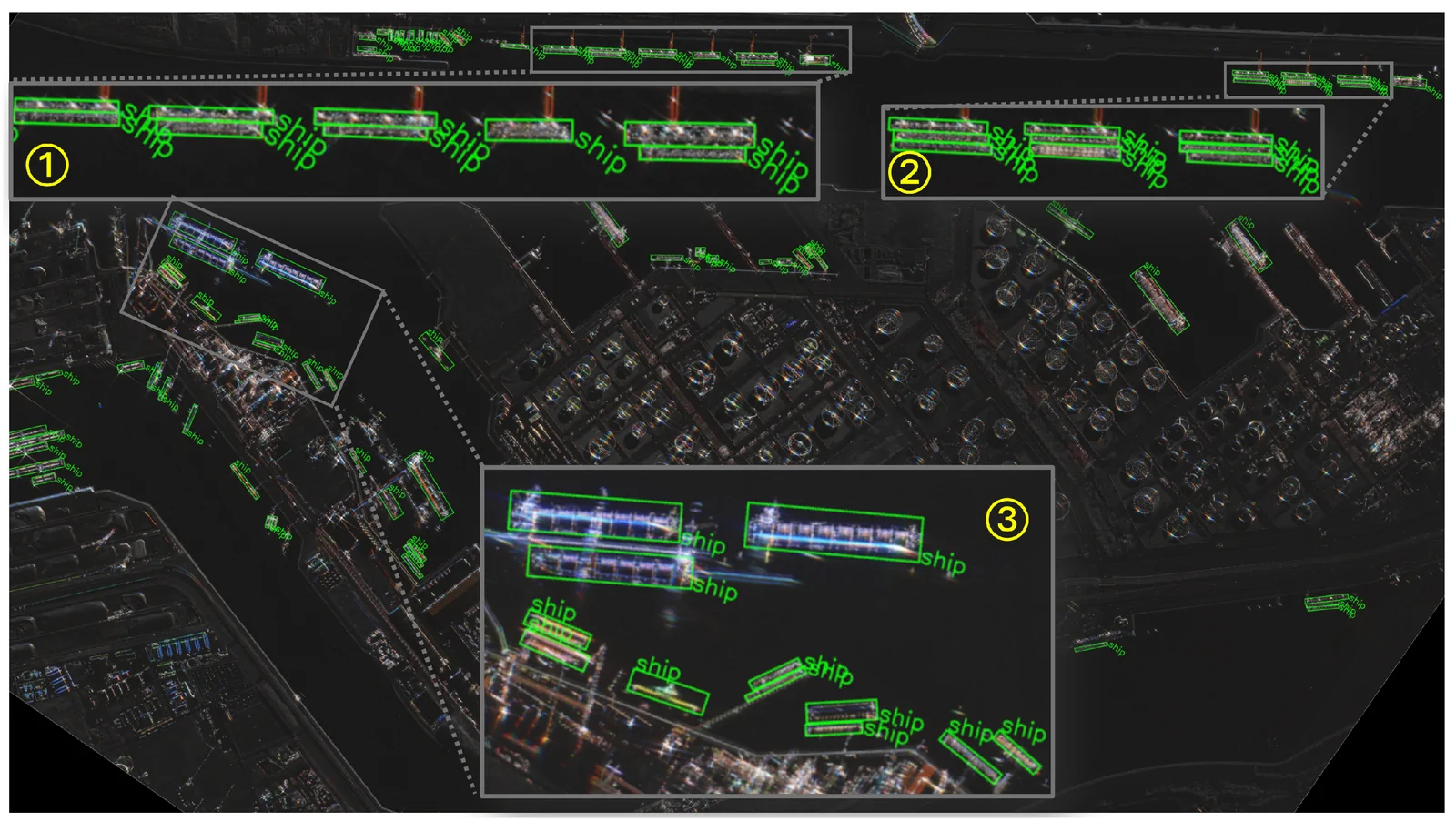
Ship annotations with CSI corresponding to Port of Rotterdam in Netherlands. Zoomed-in views of three representative regions are displayed to show the advantage of colored SAR image.

A ship detection dataset is constructed based on the ICEYE SAR satellite CSI product corresponding to Port of Rotterdam in the Netherlands. Both HBB and OBB annotations are available, with the latter illustrated in Figure 5. Three local regions are highlighted. It could be seen that with CSI it is easier to identify ships docked side-by-side at the port terminal. The large-scene image is cut into 306 image chips of size 2048×2048, and the training-test ratio is set as 7:3.

Table 1 compares performance of several representative HBB and OBB models. The former include PP-YOLOE+, PP-YOLOE [46], fully convolutional one-stage object detector (FCOS) [47], adaptive training sample selection (ATSS) [48], and task-aligned one-stage object detection (TOOD) [49], while the latter include the oriented R-CNN (O-RCNN) [50], rotated FCOS [51], region-of-interest (RoI) Transformer [52], rotation-equivariant detector (ReDet) [53] and single-shot alignment network (S2A-Net) [54]. HBB models and OBB models are pretrained with FAIR-CSAR-Ship and RSAR dataset, respectively. It can be seen that the AIS-based delimitation masks brought an average improvement of 3.42% in $mAP_{50}$ for the HBB models, with maximum improvement being 5.2%. Meanwhile, an average improvement of 4.34% in $mAP_{50}$ is observed for the OBB models, with maximum improvement being 5.7%. The PPYOLOE+ is the best HBB model for the task at hand, with $mAP_{50}$ reaching 80.5%. The RoI Transformer is the best OBB model, with $mAP_{50}$ reaching 88.4%. Table 2 illustrates the performance improvement brought by ensemble learning. It can be seen that with three-model (O-RCNN, S^2^ANET, and rotated FCOS) ensemble learning based on OWBF, $mAP_{50}$ reaches 89.0%. Moreover, with four-model (RoI Transformer, ReDet, O-RCNN, and rotated FCOS) ensemble learning, $mAP_{50}$ reaches 90.0%. The results presented in Table 2 are also included in a paper we submitted to 2026 Chinese Institute of Electronics (CIE) International Conference on Radar, which is dedicated to SAR ship detection [55].

### 5.2. Vehicle Detection Experiment

**Figure 6:**
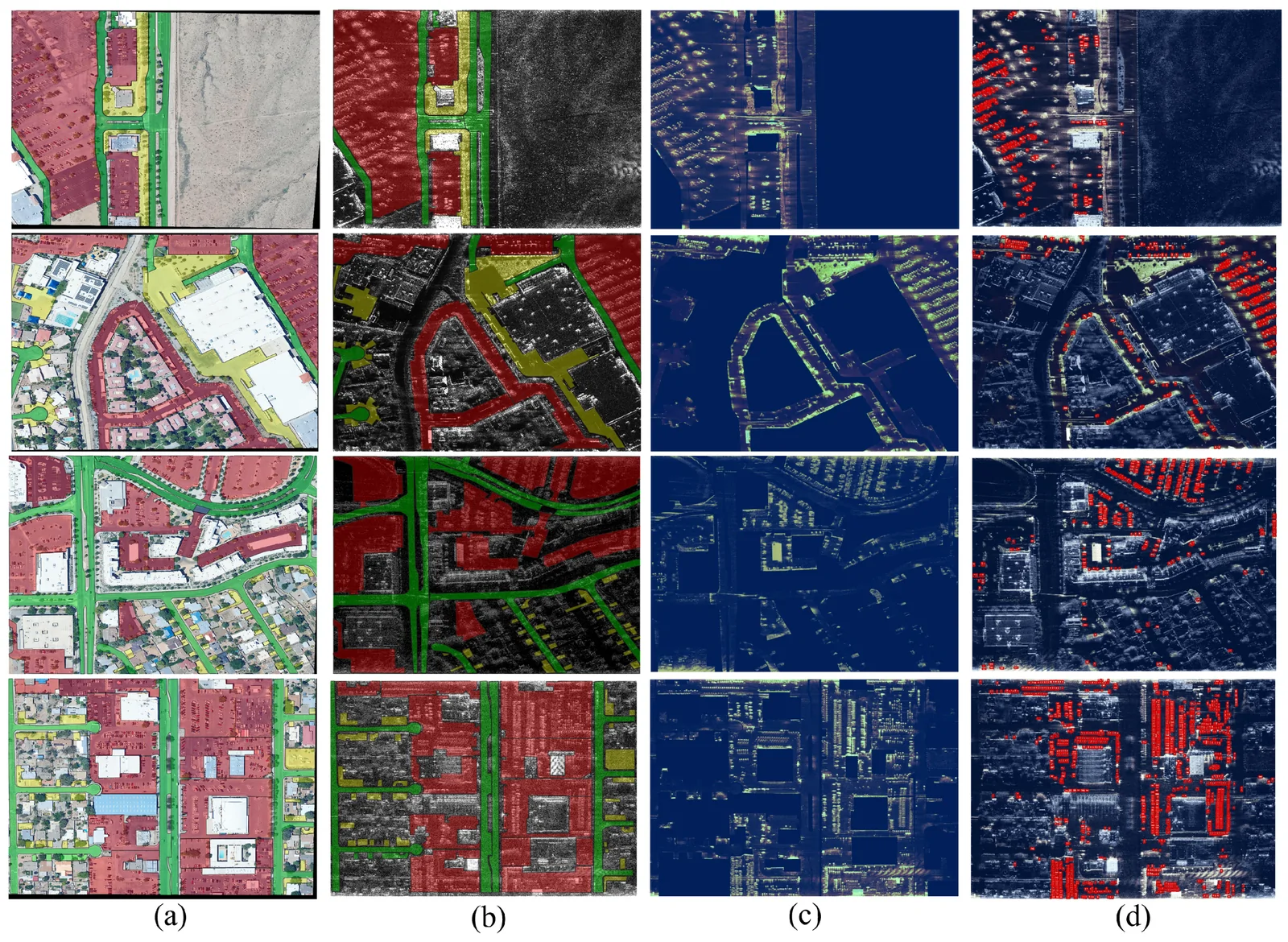
Examples of training image samples, where “Road”, “parking lot”, and “other parking” are marked by green, red and yellow, respectively. (**a**) Annotated optical image. (**b**) Annotated SAR image $I_{SAR}$. (**c**) Knowledge-aided masking image $I_{mask}$. (**d**) Context-driven color-enhanced image $I_{context}\left(\alpha =2\beta \right)$.

Leveraging the geographic data acquired from open platforms such as OpenStreetMap and GoogleEarth, we upgrade the FARAD SAR data, which contains 30 large-scene images, to a dual-modal dataset consisting of aligned optical SAR images. Since the SAR images were acquired in 2015 while the optical images were acquired very recently, some details do not match perfectly. 18 images are selected for the experiment, 9 for training and 9 for test. Figure 6a,b depict four aligned optical SAR image pairs used for training, where “road”, “parking lot”, and “other parking” are marked by green, red and yellow, respectively. The size of each SAR image is about 5736×4028. Figure 6c illustrates the knowledge-aided masking images, $I_{mask}$, which are obtained by first removing non-parking regions based on the semantic mask and then combining the outputs generated by the IDAN filter, the refined Lee filter, the improved Lee Sigma filter, and the boxcar filter as illustrated in Figure 2. Figure 6d presents the context-driven color-enhanced images used for vehicle detection, $I_{context}$, where the vehicles are indicated by red bounding boxes.

**Figure 7:**
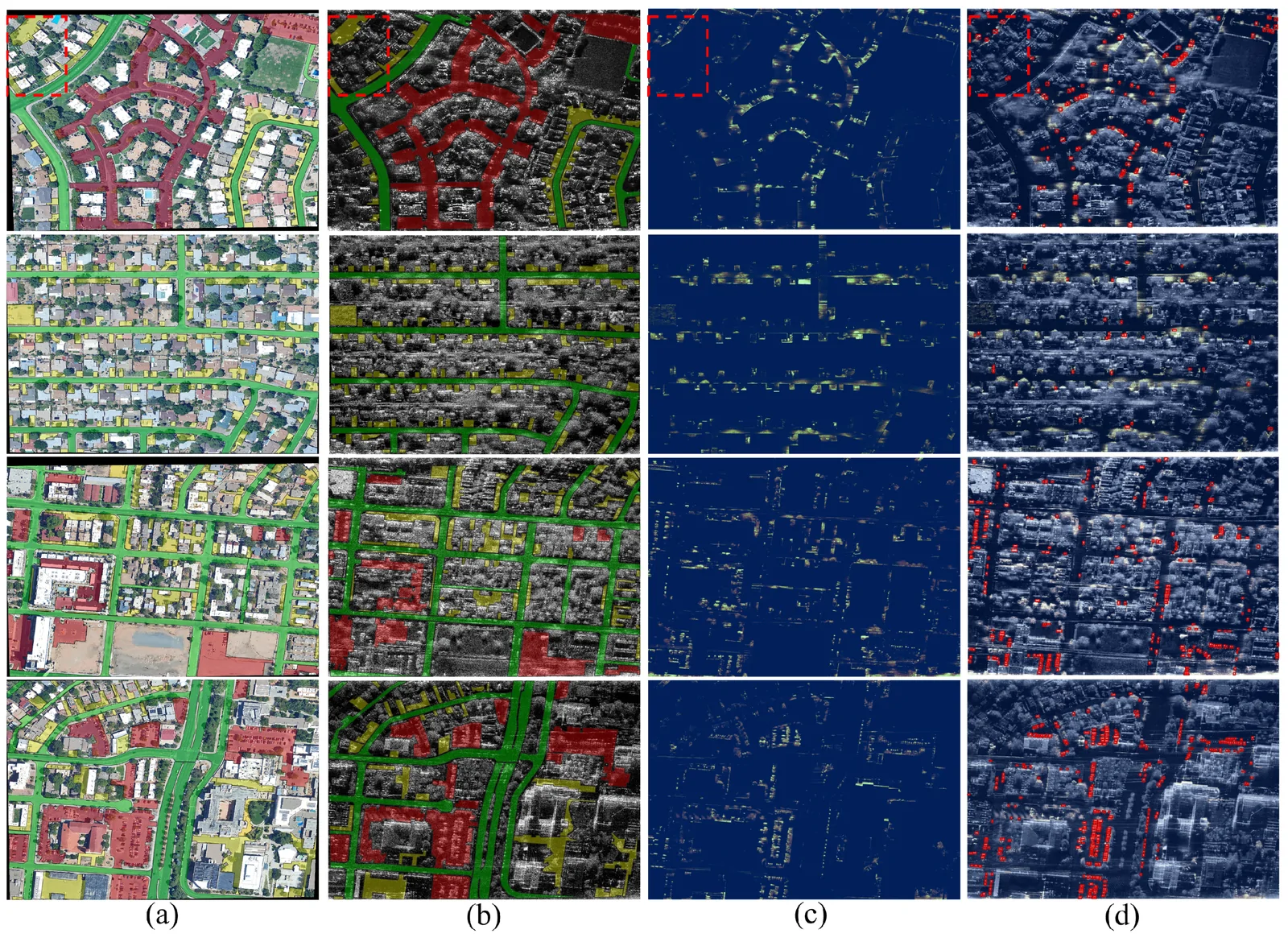
Examples of test image samples, where “Road”, “parking lot”, and “other parking” are marked by green, red and yellow, respectively. (**a**) Annotated optical image. (**b**) Annotated SAR image $I_{SAR}$. (**c**) Knowledge-aided masking image $I_{mask}$. (**d**) Context-driven color-enhanced image $I_{context}\left(\alpha =2\beta \right)$. The region indicated by the bounding box in red dashed line in the first row is analyzed in detail in Figure 8.

**Figure 8:**
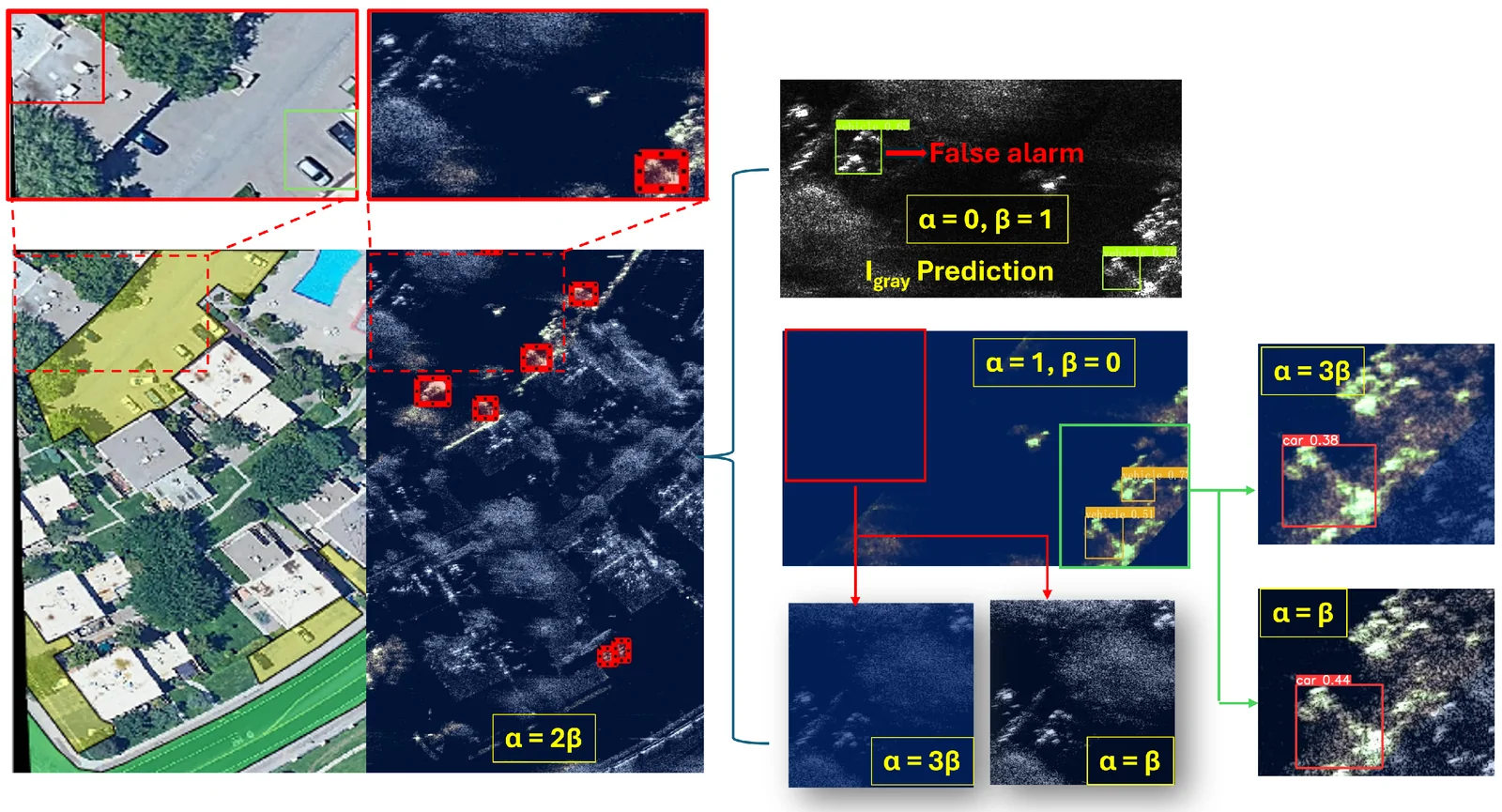
False alarm suppression with CD-SAR-REM, which corresponds to the ROI marked by red dashed lines in the first row of Figure 7 [56].

Figure 7 depicts four aligned optical SAR image pairs along with $I_{mask}$ used for test. It could be seen that the test images are very different from the training images. Suppose the region indicated by the bounding box in red dashed line in Figure 7a is of great interest, a detailed analysis is provided in Figure 8 [56]. It could be seen that the proposed CD-SAR-REM leads to higher precision and could be exploited when precision is of higher priority than recall rate.

**Figure 9:**
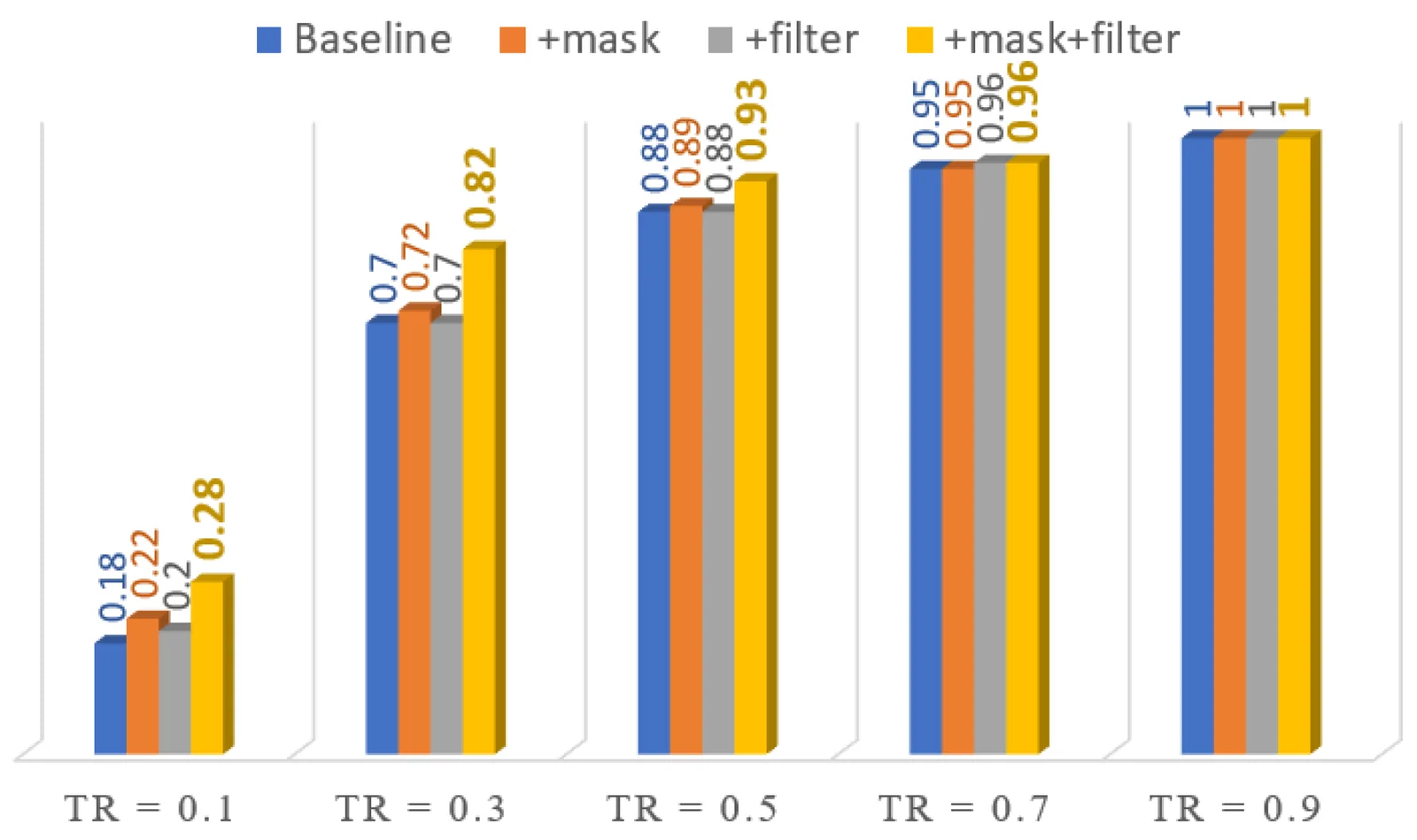
Ablation experiment for the proposed CD-SAR-REM for vehicle detection.

**Figure 10:**
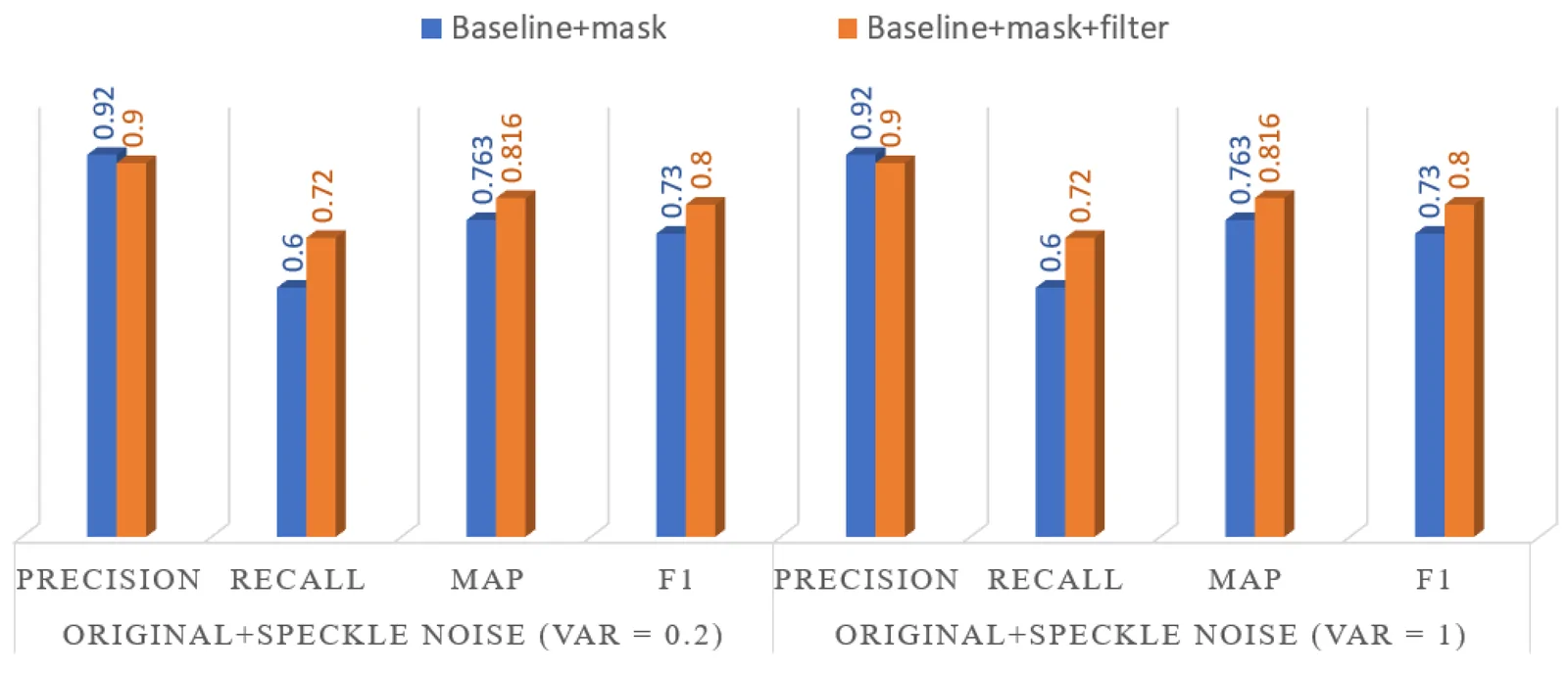
Performance improvement brought by speckle filtering in CD-SAR-REM.

To verify the individual contribution of masking and speckle filtering, results obtained in ablation experiments for the proposed CD-SAR-REM for vehicle detection are provided in Figure 9, where “TR” is the confidence score threshold. To demonstrate performance improvement brought by speckle filtering under various noise conditions, extra speckle noise of variance = 0.2 variance = 1 and are added to the original image. The results are summarized in Figure 10. It could be seen that with improvements in mAP and F1 brought by filtering increase with the variance of the extra speckle noise.

### 5.3. Aircraft Detection Experiment

#### 5.3.1. CSI-Aided Aircraft Detection with GLT

**Figure 11:**
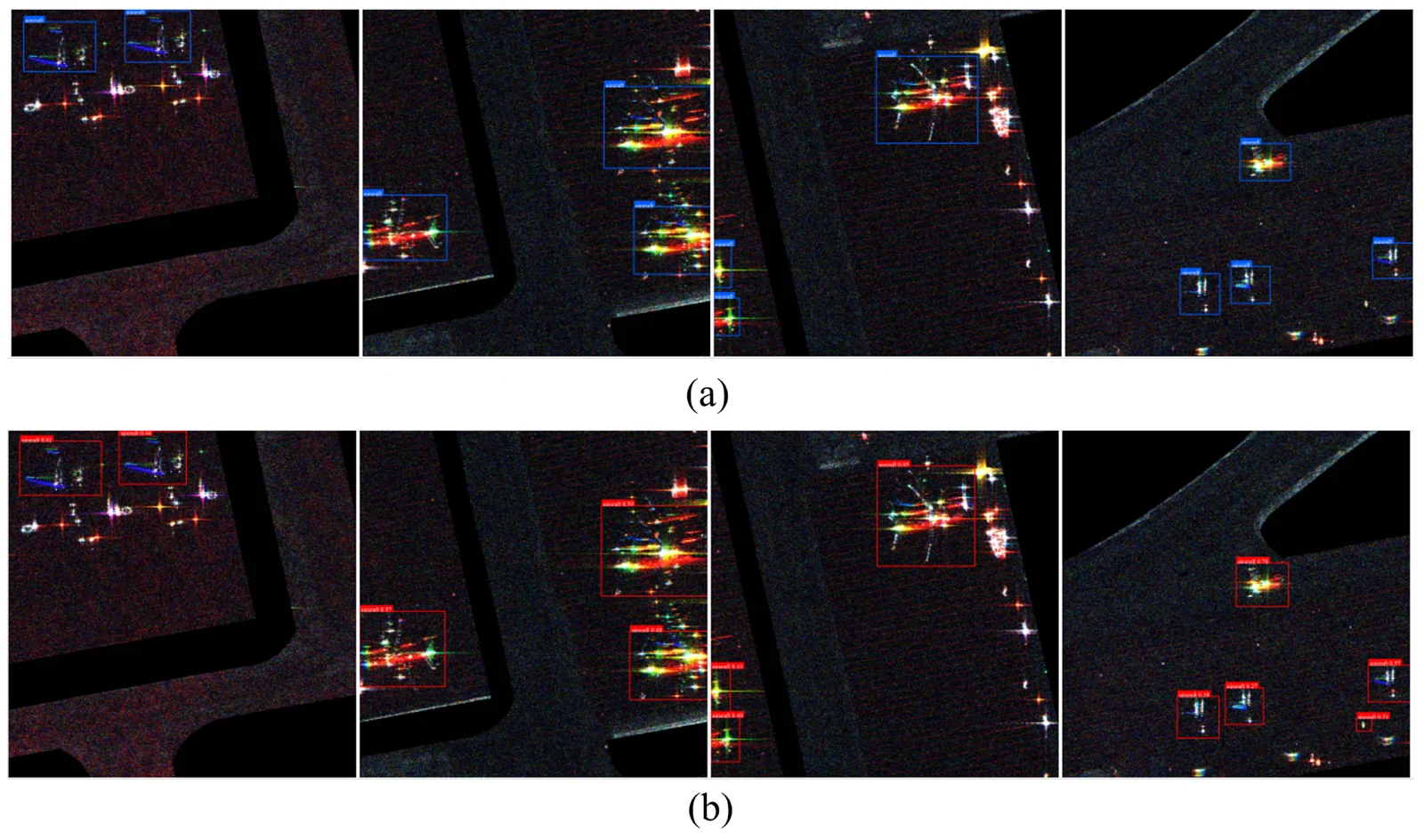
Prediction results generated by the proposed global–local–target workflow based on CSI product. (**a**) Ground truth. (**b**) Inference result.

Due to limited availability of CSI products that could support SAR aircraft detection, context-guided target detection is carried out by jointly exploiting SAR-Aircraft-1.0 and self-annotated SAR images. Specifically, the training data contains 2400 image chips (1024×1024) and 3078 instances, the majority of which are from SAR-Aircraft-1.0, while the others are cut from ICEYE satellite SAR images featuring Heathrow Airport in the U.K., Kuala Lumpur International Airport in Malaysia, Suvarnabhumi airport in Thailand, Incheon Airport in South Korea. The test data contains 1292 image chips and 159 instances, which are cut from CSI product corresponding to the Hamad International Airport in Qatar as the test data. The overlap ratio threshold $Tr_{overlap}$ is set as 0.18, the weight decay coefficient is set as 0.45, and the NMS threshold is set as 0.36. With PPYOLOE as the baseline network, performance improvement brought by OPTshare and AIS are summarized in Table 3. The ground truths and the prediction results generated by the proposed GLT workflow are illustrated in Figure 11.

#### 5.3.2. Aircraft Detection Experiment with Colored SAR Images Based on PHC

**Figure 12:**
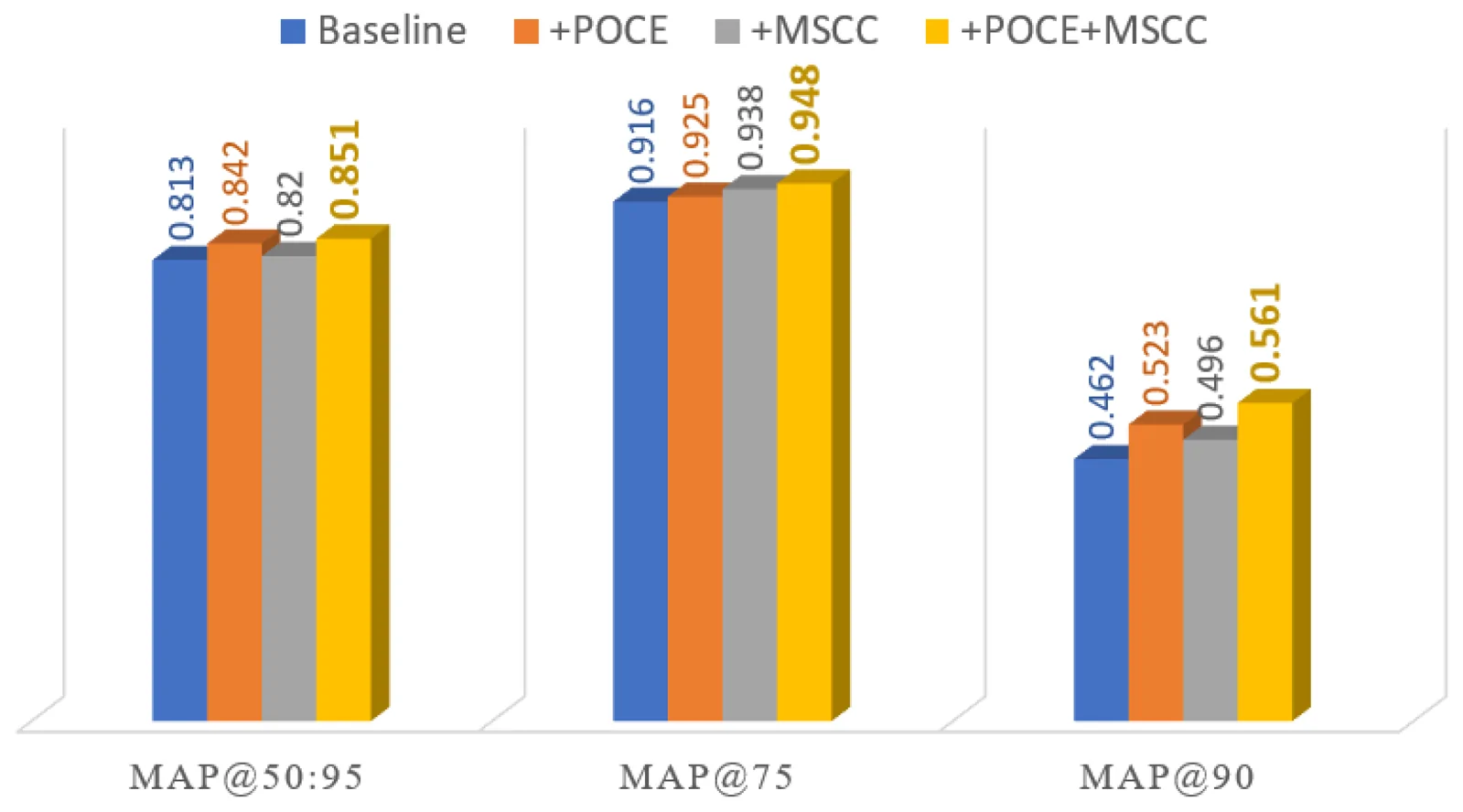
Ablation experiment for context-guided aircraft detection with colored SAR images based on PHC.

To support the experiment, a SAR aircraft detection dataset is constructed by fusing aircraft patches from SAR-Aircraft-1.0 into the satellite SAR images corresponding to 10 civil airports across the world released by Capella Space, which include Orlando Sanford International Airport (U.S.A.), Boston Logan International Airport (U.S.A.), Montego Bay Sangster International Airport (Jamaica), Van Ferit Melen Airport (Turkey), N’Djamena International Airport (Chad), Velana International Airport (Maldives), Juba International Airport (South Sudan), Panamá Pacífico International Airport (Panama), Reykjavík Airport (Iceland), and an airport in Alaska, U.S.A. The target patches from each aircraft category were divided into training and testing subsets according to a training–test ratio of 7:3 prior to the image synthesis process, and the synthesis procedure was subsequently performed independently for each subset. The dataset contains 2268 images (1024×1024) and 6801 aircraft instances. Number of instances for each category of aircraft are summarized in Table 4. The models are trained for 100 training epochs with a batch size of 2. The initial learning rate is set as 0.001, which is reduced by a factor of 0.1 at the 80th epoch and the 95th epoch. ResNet50 is used as the backbone of the proposed model. The corresponding ablation experiments are provided in Figure 12.

Table 5 compares the proposed SAR aircraft detection method with representative one-stage (TOOD, YOLOv8m), two-stage (Faster R-CNN, Cascade R-CNN), transformer-based (Deformable DETR [57], Align-DETR [58]) and SAR-specialized methods (PGD [29], SFRE-Net [26]) [45]. The advantage of the proposed method over the other methods is obvious.

**Figure 13:**
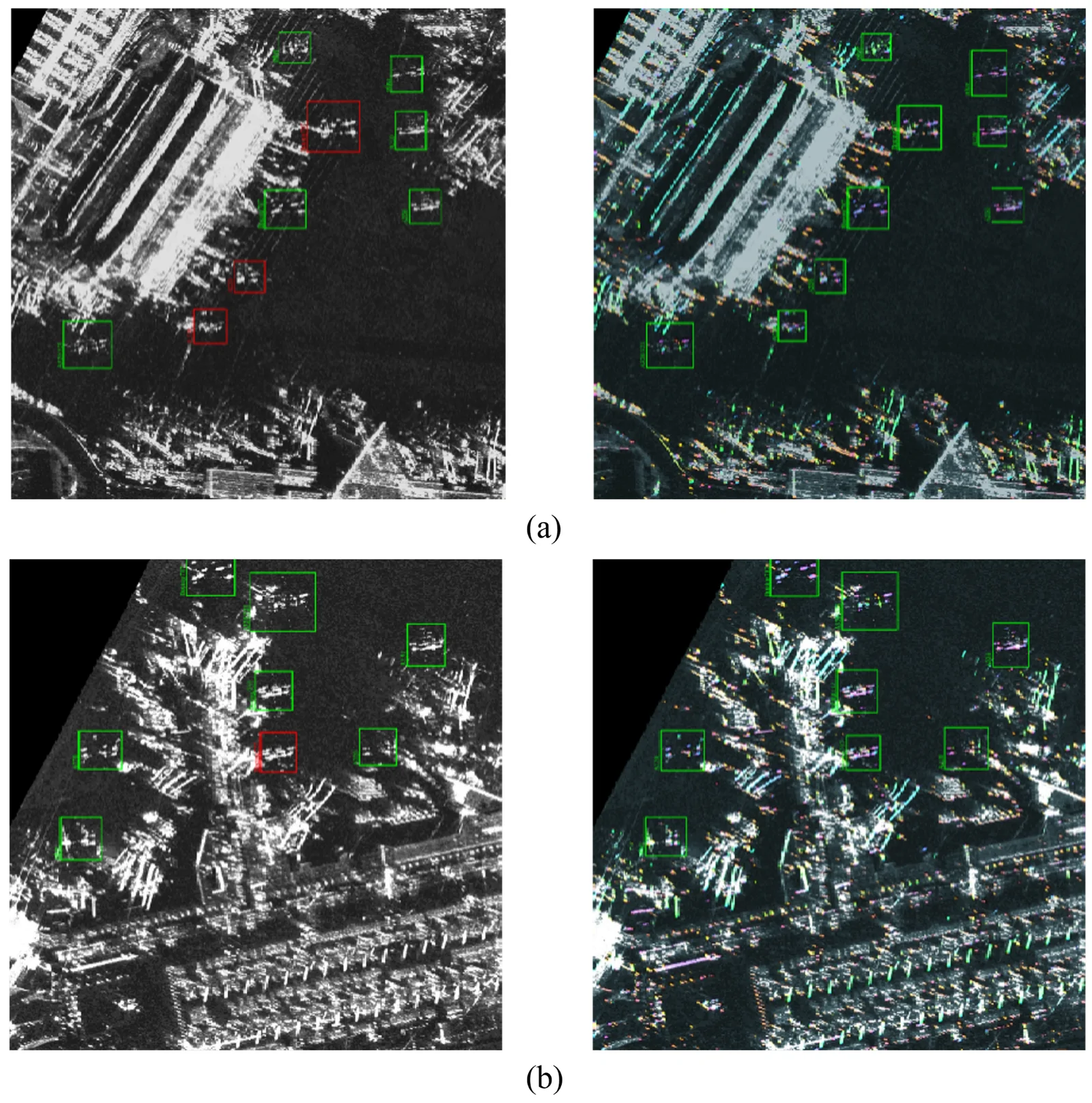
Aircraft detection results for two representative scenes obtained with the PGD and the proposed method [45]. (**a**) Scene 1. (**b**) Scene 2.

Aircraft detection results for two representative scenes obtained with the PGD and the proposed method are illustrated in Figure 13, where green and red boxes indicate an IoU greater and less than 0.8, respectively. These results are also included in the manuscript we submitted to *Remote Sensing* [45]. It could be seen that by jointly exploiting the pseudo-colored SAR images based on PHC and the contextual information extracted by the MSCC module, the proposed method achieves higher IoU than PGD.

### 5.4. Limitations

#### 5.4.1. Ship Detection

**Figure 14:**
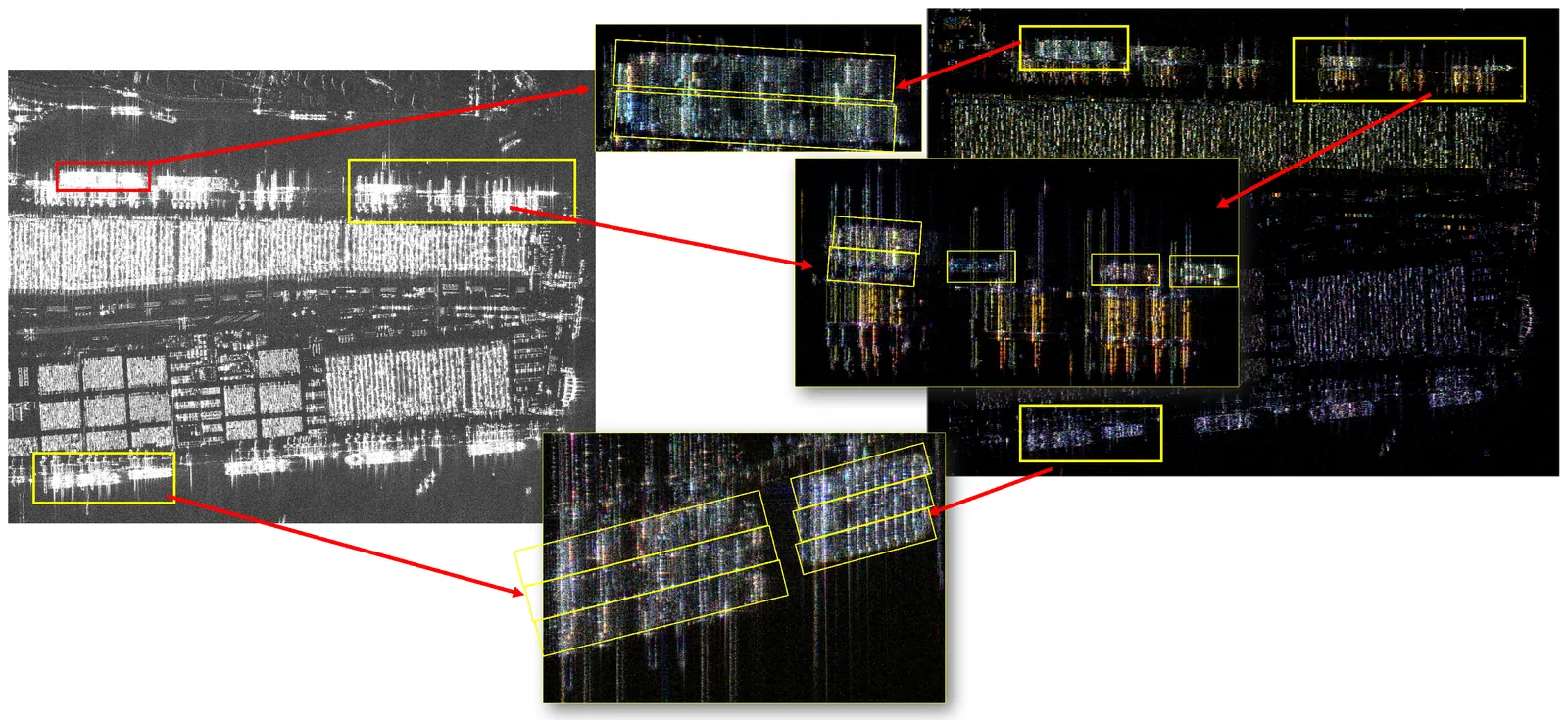
Exploiting the CSI product to identify two or three ships docked side-by-side at the port terminal. Zoomed-in views of three representative regions are provided.

An example is provided in Figure 14 to illustrate the advantage of CSI product over the grayscale SAR image in identifying two or three ships docked side-by-side at the port terminal, where the CSI product is generated with Matlab^®^ SAR Toolbox 2018 based on the Sensor-Independent Complex Data (SICD) downloaded from the ICEYE website. We admit that it is better to quantify the performance improvement statistically rather than providing individual examples. In the future, we will recruit 50–60 undergraduate or graduate students at Nanjing University of Aeronautics and Astronautics to participate in an experiment designed to study the impact of color in SAR image interpretation [59].

#### 5.4.2. Vehicle Detection

Since the performance improvement brought by mask varies greatly with the number of target-like discrete clutters in the non-parking regions in the test images, it is not quantified. Unlike the experiment for ship and aircraft detection, where the test image are the CSI products for Port of Rotterdam and Hamad International Airport in Qatar, respectively, partition of training and test images of FARAD dataset make a huge difference on this performance metric. It is easy to see from Figure 6 and Figure 7 that the parking patterns are much more complicated than the land–water segmentation task for ship detection and runway/apron segmentation task for aircraft detection, and the performance improvement brought by mask varies greatly if the training and test images switch roles. Moreover, although some readers might argue that the layout of the parking region is not always known, based on commonsense reasoning false alarms in non-parking regions would be suppressed once the information is known.

#### 5.4.3. Aircraft Detection

The synthetic SAR image dataset used in Section 5.3.2 is like the MixMSTAR dataset constructed by Liu, et al. from the Rocket Force University of Engineering [60]. Both the targets and the background are real but were collected from different angles at different time. Realistic scenes are synthesized by inserting the targets into backgrounds based on real-world operational rules. Nevertheless, we admit that the synthesized samples are over-ideal and cannot truthfully represent the volatile nature of real SAR images. Even in SAR images collected with the same imaging geometry, SAR signatures of the same aircraft/helicopter could be different due to the complicated scattering mechanism. Passenger Boarding Bridge (PBB) and other nearby facilities could have great impact on SAR signature of aircraft, and the blades could always get in the way for helicopters. Therefore, the generalization capability of PACE-Det under different sensors, imaging conditions, and real-world airport environments requires further investigation.

#### 5.4.4. Generalisation Problem

It has been shown in many studies that incorporating long-term multi-modal contextual information could facilitate SAR target detection in complex backgrounds. For instance, Ai Jiaqiu et al. from Hefei University of Technology proposed integrating feature maps derived from long-term automatic identification system data with real-time SAR images to enhance sea–land segmentation [61,62], which resonates strongly with the essential idea of this study. Although limited data from a small number of locations are used in the experiments in the manuscript, the idea of using map to bridge the gap between SAR and optical images could generalise to other ports, airports, sensors, and acquisition conditions via map-guided multimodal knowledge distillation. Once a teacher model is obtained by learning the operation rules from multimodal data, a single-modal student network can work normally in SAR-only cases, just like humans do not need to see all the airports to know that aircraft park on apron that follows specific design rules (and that is how the human annotators make accurate annotations based on SAR image only). In the future, we plan to refine the proposed method with an expanded version of the data used in this manuscript and datasets constructed by other researchers in the field, e.g., the multimodal salient ship detection (MSSD) dataset containing 12920 SAR automatic identification system (AIS) ground truth (GT) triplets [63].

## 6. Conclusions

Color feature-aided SAR ship detection, vehicle detection, and aircraft detection are considered in this work. To deal with complex inshore background, CSI products are exploited for accurate ship annotations, and CGEL strategy is proposed for ship detection, which incorporates transfer learning based on benchmark SAR ship detection datasets, AIS-based land–water masking, and ensemble learning with two-stage, anchor-free and refine-stage OBB models based on OWBF. Taking into account the fact that satellite SAR CSI products can rarely be used to support vehicle detection, and those can be used to support airplane detections are also rare, alternative color-coding methods are proposed to support SAR vehicle and airplane detection. Specifically, CD-SAR-REM is proposed to generate a context-driven color-enhanced version of the original SAR image by combining the knowledge-aided masking image, which is obtained by fusing color-coded images corresponding to potential parking regions generated by diverse speckle filters, with the original SAR image based on AIS. Two methods for color feature-aided aircraft detection are presented. In case that CSI products are available, context-guided plane detection is carried out based on GLT workflow, which includes SAR optical image registration, semantic segmentation of runway and apron, context-guided masking based on AIS, and aircraft detection based on OPTshare. Otherwise, pseudo-color SAR images are generated based on PHC and fed into the core detection network to generate the initial predictions, which are further refined based on the contextual information extracted by the post-processing MSCC module. Experiments based on both the benchmark ship, vehicle and aircraft detection datasets and the self-constructed SAR datasets show that by incorporating context information and color feature into the target annotation and detection process, false alarms caused by clutters in complex background can be effectively mitigated while the precision, recall, and F1 score improve significantly.

## Figures and Tables

**Table 1:** Performance comparison of HBB and OBB models for SAR ship detection.

Paradigm	Model	${\mathrm{mAP}}_{50}$ (%)		
		Mask	Nomask	Gain
HBB	PPYOLOE+	80.5	78.2	2.3
	PPYOLOE	78.3	73.1	5.2
	TOOD	78.1	74.8	3.3
	ATSS	77.6	74.7	2.9
	FCOS	79.3	75.9	3.4
OBB	S^2^ANET	85.4	80.5	4.9
	RoI-Transformer	88.4	83.8	4.6
	O-RCNN	87.2	81.5	5.7
	ReDet	88.0	85.5	2.5
	Rotated-FCOS	75.1	71.1	4.0

**Table 2:** Performance improvement brought by ensemble learning in SAR ship detection.

	S^2^ A-NET	RoI-Transformer	O-RCNN	ReDet	Rotated-FCOS	Fusion
${\mathrm{mAP}}_{50}$	0.854	0.884	0.872	0.880	0.751	0.90
**Precision**	0.848	0.916	0.893	0.951	0.802	0.967
**Recall**	0.856	0.883	0.829	0.865	0.766	0.862
**F1**	0.852	0.899	0.860	0.906	0.783	0.911

**Table 3:** Performance improvement brought by OPTshare and AIS with PPYOLOE as the baseline network. Top performer in each column is highlighted with bold font.

Method	${\mathrm{mAP}}_{25}$	${\mathrm{mAP}}_{50}$	Precision	Recall	F1
Baseline	66.5%	61.5%	73.4%	57.2%	64.3%
+OPT	70.3%	66.1%	78.9%	63.5%	70.4%
+OPT + AIS (α = 0.75)	72.6%	67.2%	**80.0%**	**67.9%**	**73.5%**
+OPT+ AIS (α = 1)	**72.7%**	**68.8%**	**80.0%**	65.4%	72.0%

**Table 4:** Number of instances for each category of aircraft in the experiment.

Category	A220	A320/321	A330	ARJ21	Boeing737	Boeing787	Other
**Instances**	909	905	902	904	906	911	1364

**Table 5:** Advantage of the proposed SAR aircraft detection method over the mainstream methods [45]. Performance of the proposed method is highlighted with bold font.

Category	Method	${\mathrm{mAP}}_{50:95}$	${\mathrm{mAP}}_{90}$
One-Stage	TOOD	0.815	0.411
	YOLOv8m	0.835	0.462
Two-Stage	Faster R-CNN	0.784	0.408
	Cascade R-CNN	0.802	0.417
Transformer	Deformable DETR	0.776	0.357
	Align-DETR	0.817	0.396
SAR-Specialized	PGD	0.790	0.392
	SFRE-Net	0.753	0.355
**SAR-Specialized**	**Proposed**	**0.851**	**0.561**

## Data Availability

Some of the data are available upon request.
